# Memoirs of the Royal Academy of Sciences at Paris

**Published:** 1781-10

**Authors:** 


III. *Memoirs of the Royal Academy of Sciences at Paris.* *(Continued from page* 110.*)*

XX. *OBfervations on nitre with an abforbent earthy bafis procured from the faltpetre of rubbifh. By* M. Sage.—This writer gives the name of abforbent earth to that which is found in the bones after calcination, when the *natrum* they contained is feparated by wafhing; and he has obferved, that nitre with a non-alkaline bafis *(nitre à bafe non alkaline)* which is procured from the faltpetre of rubbifh, has a greater re-

 femblance femblance to that formed with this earth of the bones, than to nitre with a bafis of calcareous earth.

XXI. *An account of a fingular Aurora Borealis obferved at Paris February* 26, 1777, *and of two other remarkable ones on Nov.* 3, *and Dec.* 3 *of the fame year By* M. Meffier.

The author has added accounts of thefe phæ-nomena as obferved at Nancy, Caen, Berlin, the Hague, Amfterdam, Franeker, Vienna, and other places.

XXII. *Experiments on the Cold of* 1776. *By* M. M. Bezout, Lavoifier, and Vandermonde.

The cold at Paris appears to have been two degrees (of de Reaumur) greater in 1776 than it was in 1709.

XXIII. *Obfervations on the refrigerant power of liquors, as well fimple as compound. By* M. M. Cadet and Briffon.

XXIV. *Obfervations on combuftion in general. By* M. Lavoifier.—The theory of combuftion, which this ingenious philofopher attempts to eftablifh, is briefly as follows: he confiders vital air, as he terms it, or what Dr. Prieftley would call dephlogifticated air, as being formed of a certain bafis combined with the igneous fluid. This bafis he fuppofes to have a greater affinity to to the body that is burnt, or ſome of its principles, than to the igneous fluid; it is therefore diſengaged from it, and the igneous fluid ſeparates in the form of flame; ſo that when a ſubſtance is ignited, it is not that which burns, but the air around it.

M. Lavoiſier remarks, that in the combuſtion of bodies four phænomena are conſtantly obſerved. 1. There is a diſengagement of the matter of fire or light. 2. Bodies can burn only in a ſingle ſpecies of air, the vital air juſt now ſpoken of. 3. There is a decompoſition of this vital air, and the burnt body increaſes in weight in proportion to the quantity of air deſtroyed. 4. The body that is burnt is changed into an acid, by the addition of the ſubſtance which has increaſed its weight; ſo that if ſulphur is burnt under a bell, the reſult is vitriolic acid; if phoſphorus, the acid of phoſphorus: if charcoal, fixed air, or the chalky aeriform acid. He conſiders the calcination of metals as being ſubmitted to the ſame laws, but with this difference, that inſtead of an acid, we have a particular combination, a metallic calx. He obſerves, that theſe different phænomena admit of an eaſy explanation by Stahl's hypotheſis, which ſuppoſes phlogiſton to be in a fixed ſtate in metals,

ſulphur, fulphur, and all other combuftible bodies, but if one of Stahl's followers were to be called upon to prove its exiftence, he muft neceffarily reply, that combuftible bodies contain the matter of fire becaufe they burn, and that they burn becaufe they contain the matter of fire. This, fays our author, would be explaining combuftion by combuftion; he therefore confiders the exiftence of this matter of fire, this phlogifton, in metals, fulphur, &c. as being in reality altogether imaginary. If afked, what he means by the matter of fire? He anfwers with Franklin, Boerhaave, and fome of the ancients, that the matter of fire, or light, is a very fubtile, rare, elaftic fluid, which furrounds the planet we inhabit, and penetrates with greater or lefs facility the different bodies that compofe it.

Towards the clofe of his paper, the author prefents us with a theory of animal heat. He fuppofes that the vital air received by the lungs is there decompofed, and that what is expired is fixed air. This decompofition, we are told, takes place, becaufe the bafis of the vital air is feparated from the igneous fluid, and the latter being thus difengaged produces heat.

XXV. *Remarks on certain difeafes of the liver, which are attributed to other organs; and on dif-*

eafes,

*cases, the seat of which is commonly though improperly supposed to be in the liver. By* M. Portal.—In feeling the liver it is often found to extend lower than in its natural ſtate, and the phyſician erroneouſly conſiders it as the ſeat of the diſeaſe, when only the right lobe of the lungs is affected. M. Portal relates ſeveral caſes in which he himſelf was miſtaken in this manner. He obſerves likewiſe, that the ſpleen may be affected in a ſimilar way, by the left lobe of the lungs. In the late Duke de Chaulnes, M. de Bordeu and our author felt a tumour under the falſe ribs, which they ſuppoſed to be the liver, but on diſſection that viſcus was found to be perfectly ſound.

M. Portal takes occaſion to notice the jaundice of new-born infants, an affection which he ſuppoſes to be owing, not to any diſeaſe of the liver, but merely to the bile inſinuating itſelf into the lacteals. Upon paſſing a ligature round the ſmall inteſtines of dogs, a little below the opening of the ductus choledochus, he has obſerved, that in five or ſix hours the tunica conjunctiva of their eyes has acquired a yellow tinge, and their lacteals have been found filled with bile.

If

If the liver is ſometimes ſuſpected without reaſon, it is often, on the other hand, the unſuſpected ſeat of the diſeaſe. Our author mentions two caſes in which this happened. One of theſe patients was a woman, who complained of pain in the epigaſtric region, attended with vomiting. The diſorder was treated with bitters and ſtomachics, but without any good effect, as the patient died of hectic fever. On diſſection the ſtomach was found to be in a healthy ſtate, but the liver was enlarged and diſeaſed. Some years afterwards he applied this obſervation to the caſe of the Marchioneſs d'Epagny, who had been treated for a ſtomach complaint. She had vomitings, cough, dyſpnœa, ſwelled legs, and hectic fever. Upon examination he found the liver enlarged, and by means of kermes mineral given in ſmall doſes, with terra foliata tartari, the patient recovered.

He next treats of hæmorrhage from the ductus choledochus, and mentions one caſe in which it proved fatal; but he ſays in general it is ſo far from being dangerous, that it is uſeful by preventing inflammation. He ſpeaks of a ſtudent, who, after having experienced inflammation and pain in the region of the liver, was relieved by diſcharging a great quantity of blood blood by vomit and ftool. He likewife relates the cafe of M. Aublet, a celebrated botanift, who had an enlargement of the fpleen, and was carried off by a profufe hæmorrhage from the mouth and anus. On diffection the fpleen was found to be as large as a child's head, and the veffels between the fpleen and the ftomach were dilated, and ftill pouring blood into the cavity of the ftomach.

XXVI. *Botanico-meteorological Obfervations, made at the caftle of Denainvilliers near Pithiviers in Gatinois in* 1776. *By* M. du Hamel.

XXVII. *Third memoir on different fubjects of Natural Hiftory and Chemiftry. By* M. Montet.

This paper relates to the mineralogy of a part of the Cevennes.

