# Errata

**Published:** 1781-12

**Authors:** 


					p. ?27, 1. 19, fo>
' M. Sag?* read ' M, Cadet.

				

